# Characterizing the prognostic and therapeutic value of necroptosis in sarcoma based on necroptosis subtypes

**DOI:** 10.3389/fgene.2022.980209

**Published:** 2022-09-27

**Authors:** Yibo Ma, Qihang Yuan, Shiping He, Xiulin Mao, Shuo Zheng, Changjian Chen

**Affiliations:** ^1^ Graduate School of Dalian Medical University, Dalian Medical University, Dalian, China; ^2^ Department of General Surgery, First Affiliated Hospital of Dalian Medical University, Dalian, China; ^3^ Department of Orthopedics, Subei People’s Hospital Affiliated to Yangzhou University, Yangzhou, China; ^4^ Department of Anesthesiology, Dalian Municipal Central Hospital, Dalian, China; ^5^ The Second Ward of Department of Orthopedics, The Second Hospital of Dalian Medical University, Dalian, China; ^6^ The First Ward of Department of Orthopedics, The Second Hospital of Dalian Medical University, Dalian, China

**Keywords:** necroptosis, sarcoma, subtypes, immune infiltration, prognosis, bioinformatics analysis

## Abstract

Necroptosis, a type of necrotic cell death independent of caspase regulation, is mainly mediated by receptor interacting serine/threonine kinase 1 (RIPK1), receptor interacting serine/threonine kinase 3 (RIPK3) and mixed lineage kinase domain-like (MLKL). Necroptosis plays an essential role in many tumors. However, the potential roles of necroptosis in tumor microenvironment (TME) of sarcoma (SARC) remain unknown. This study analyzed the expression, prognosis, genetic alterations of necroptosis genes in SARC. We identified two subtypes (cluster A and B) by performing unsupervised consensus clustering. Cluster A and B greatly differed in prognosis and immune infiltration, with cluster A showing more favorable prognosis, higher immune infiltration and higher expression levels of necroptosis genes than cluster B. Based on the differentially expressed genes (DEGs) between two clusters, a necroptosis scoring system was developed for predicting overall survival of SARC patients. Patients with high necroptosis score had worse survival status, with a decreased infiltration level of most immune cells. Our findings demonstrated the potential role of necroptosis in regulating tumor microenvironment and the prognostic value of necroptosis-related genes for SARC patients.

## Introduction

Tumor cells may occur in different organizations, usually from the epithelial tissue of the malignant tumor called cancer, from the mesenchymal tissue of origin and the malignant tumor called sarcoma. Soft tissue refers to the non-epithelial bone tissue of the body. Soft tissue sarcoma is a heterogeneous malignancy that includes more than 60 different diagnoses. Despite their wide variety, soft tissue sarcomas are rare, accounting for less than 1% of all adult cancers. Since the 1990s, improved surgery at specialized centers, combined with preoperative or postoperative radiotherapy or chemoradiotherapy, has improved outcomes for patients with localized disease. However, despite the success of initial treatment, about 50% of patients relapse, often with long-term failure (Karavasilis et al., 2008).

Apoptosis, which is an autonomous and orderly death of cells, involves the activation, expression and regulation of a series of genes, and is also known as programmed cell death (PCD). Typical apoptosis pathways include endogenous mitochondrial pathway, endoplasmic reticulum pathway and exogenous death receptor pathway (Cetraro et al., 2022) (D'Arcy, 2019). In recent years, necroptosis as a new type of PCD has been proposed, opening a novel field of apoptosis.

Necroptosis is a kind of PCD different from the typical apoptotic pathway (Gong et al., 2019). As we know, tumour necrosis factor (TNF)-α acts through a classical signaling pathway initiated by binding to its receptor TNFR1 (Wu et al., 2021). Classical apoptosis depends on the activation of caspase. When caspase is deficient or inhibited, and the classical apoptosis pathway is inhibited, necroptosis can be activated as a substitute for apoptosis (Gong et al., 2019) (Martens et al., 2021). In recent years, the mechanism of necroptosis is still controversial. Necroptosis is mainly mediated by receptor interacting serine/threonine kinase 1 (RIPK1), receptor interacting serine/threonine kinase 3 (RIPK3) and mixed lineage kinase domain-like (MLKL) (Zhu et al., 2019) (Qin et al., 2019). Evidence have revealed the important role of necroptosis in cancer development of various cancer types (Gong et al., 2019). For example, decreased RIPK3 expression was found to be associated with poor prognosis in breast cancer and colorectal cancer (Koo et al., 2015) (Moriwaki et al., 2015). Low expression level of MLKL was significantly correlated with shorter overall survival of gastric cancer (Ertao et al., 2016), ovarian cancer (He et al., 2013), and cervical squamous cell cancer (Ruan et al., 2015). Thus, MLKL was considered a potential prognostic biomarker. However, necroptosis is still not well studied in SARC currently. A study reported that a prognostic model based on necroptosis-related genes could predict immune characteristics and prognosis for soft tissue sarcomas (Qi et al., 2022). Binfeng Liu *et al.* comprehensively assessed the prediction value of necroptosis lncRNAs signature in soft tissue sarcomas (Liu et al., 2022).

In our study, we comprehensively evaluated the expression, genetic alteration, prognosis of necroptosis genes in SARC. Furthermore, based on the expression of necroptosis genes, we performed unsupervised clustering and identified two subtypes (cluater A and B) of SARC patients. Interestingly, the survival status of SARC patients in the two clusters were significantly different. We further explored the difference between the two clusters, including clinical features, differentially expressed genes (DEGs), pathways, and immune cell infiltration. We also constructed the necroptosis score, which could predict the overall survival of SARC patients. The correlation between necroptosis genes and the efficacy of anti-tumor drugs were also explored.

## Materials and methods

### Data collection

The expression and clinical data of TCGA-SARC (N = 265) were downloaded from UCSC-Xena database (https://xenabrowser.net/datapages/). The GSE21257 (N = 53) was obtained from Gene Expression Omnibus (GEO) database (https://www.ncbi.nlm.nih.gov/geo/). R packages “limma” and “sva” were used to eliminate batch effect. The gene set of necroptosis were downloaded from Molecular Signature Database (MsigDB, http://www.gsea-msigdb.org/gsea/index.jsp), and eight genes (FADD, FAS, FASLG, MLKL, RIPK1, RIPK3, TLR3, TNF) were enrolled.

### Online analysis

(1) The protein-protein interaction network (PPI) of necroptosis genes was constructed using STRING database (https://cn.string-db.org/). (Cetraro et al., 2022) The genetic alteration of necroptosis genes, including single nucleotide variant (SNV) and copy number variant (CNV), were analyzed using GSCA database (http://bioinfo.life.hust.edu.cn/GSCA/#/). (D'Arcy, 2019) The correlation of necroptosis genes with IC50 of anti-tumor drugs were explored using pRRophetic (Geeleher et al., 2014) in GSCA database.

### Consensusclusterplus

Base on necroptosis genes associated with prognosis, molecular subtypes were performed separately for merged data samples via the Consensus Cluster Plus 1.52.0 (Wilkerson and Hayes, 2010). “Pam” arithmetic and “pearson” distance were utilized to complete 500 bootstraps with every bootstrap having specimens (≥80%) of merged data. Cluster number k was between 2 and 10, and the optimum k was identified as per cumulative distribution function (CDF) and CDF Delta area.

### Enrichment analysis

The pathways used in Gene Set Variation Analysis (GSVA) were downloaded from MsigDB, including HALLMARY pathways, Kyoto Encyclopedia of Genes and Genomes (KEGG) pathways and Reactome pathways. The ssGSEA function in R package “GSVA” was used to calculate the pathway score of each sample. The R package “clusterprofiler” was used to perform Gene Ontology (GO) and KEGG analysis of DEGs.

### Assessment of immune cell infiltration

The ssGSEA function of R package “GSVA” was used to assess the infiltration level of 23 immune cells (Charoentong et al., 2017). The marker genes of each immune cells were shown in Supplementary Table S1. The immune cell level was further compared in the indicated groups.

### Construction of necroptosis score

The 316 DEGs (|log2Fold Change (FC)| > 0.5, *p* < 0.05) between the two clusters were identified using R package “limma”. Further univariate regression analysis identified 173 DEGs related to overall survival (*p* < 0.05). Finally, 173 DEGs determined to conduct principal component analysis (PCA), and principal component 1 and 2 were extracted to develop the necroptosis score (Yang et al., 2021). The expression profiles of genes with significant prognosis were used as input data sets, and the R software package PRCOMP was used for PCA analysis to obtain the first and second principal components. Furthermore, the first and second principal components were used as independent variable coefficients to calculate the risk score of each patient. The surv_cutpoint function of R software package SurvMiner was used to obtain the best cut-off value, and the patients were divided into high risk and low risk groups.

### Prognostic analysis

R (4.1.1) package “survival” and “survminer” were used to conduct Kaplan-Meier, univariate and multivariate regression analyses of necroptosis genes in SARC. Further nomogram model was established using R package “rms”.

### Statistical analysis

Statistical analysis was performed in R software (4.1.1). R packages and tools used in the study were indicated. Statistical methods were described in the corresponding sections. *p* < 0.05 was considered as significant. ns, no significance. *p < 0.05, **p < 0.01, ***p < 0.001, ****p < 0.0001.

## Results

### Genetic alteration of necroptosis genes

**FIGURE 1:**
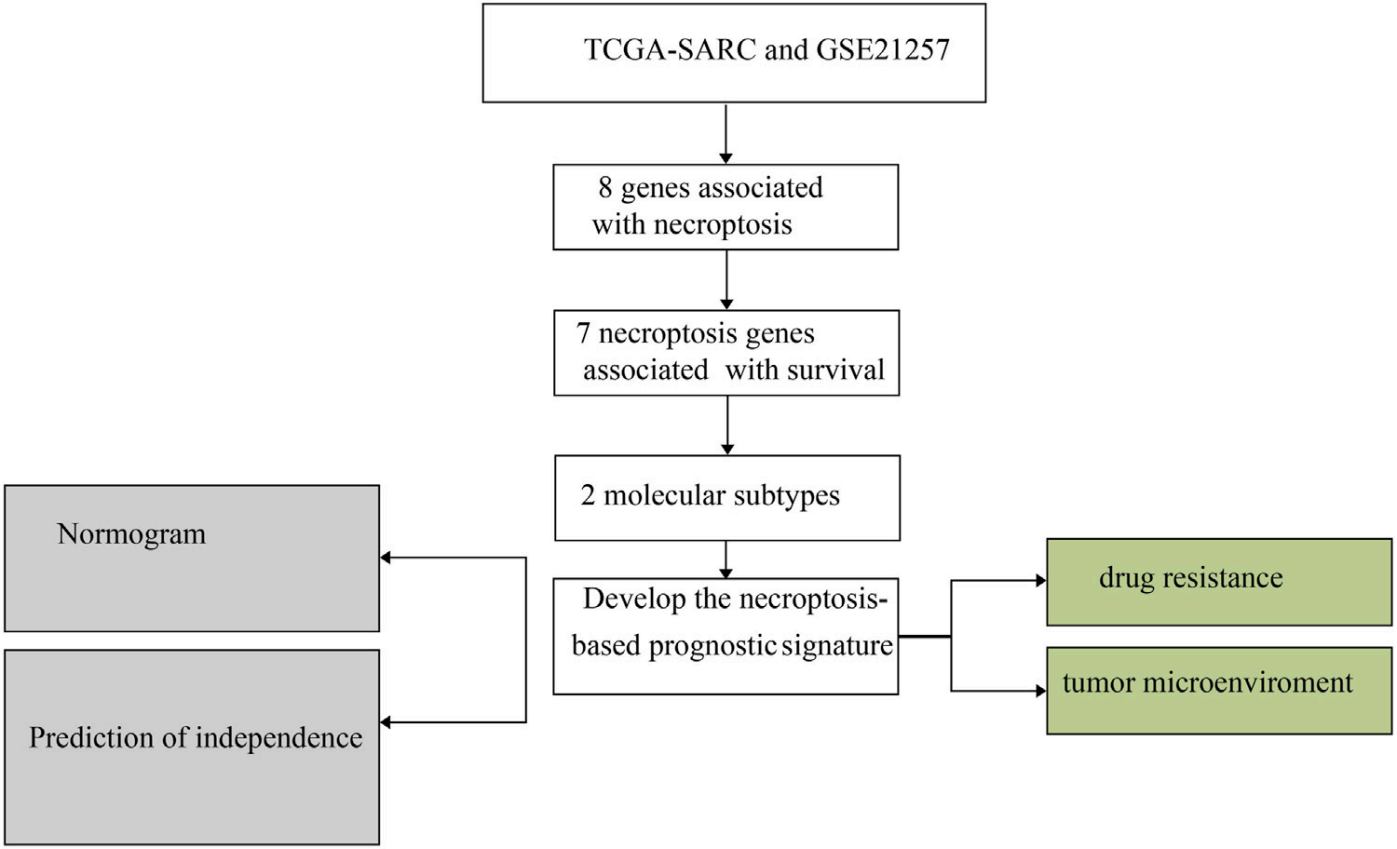
The diagrammatic sketch of necroptosis pathway.

**FIGURE 2:**
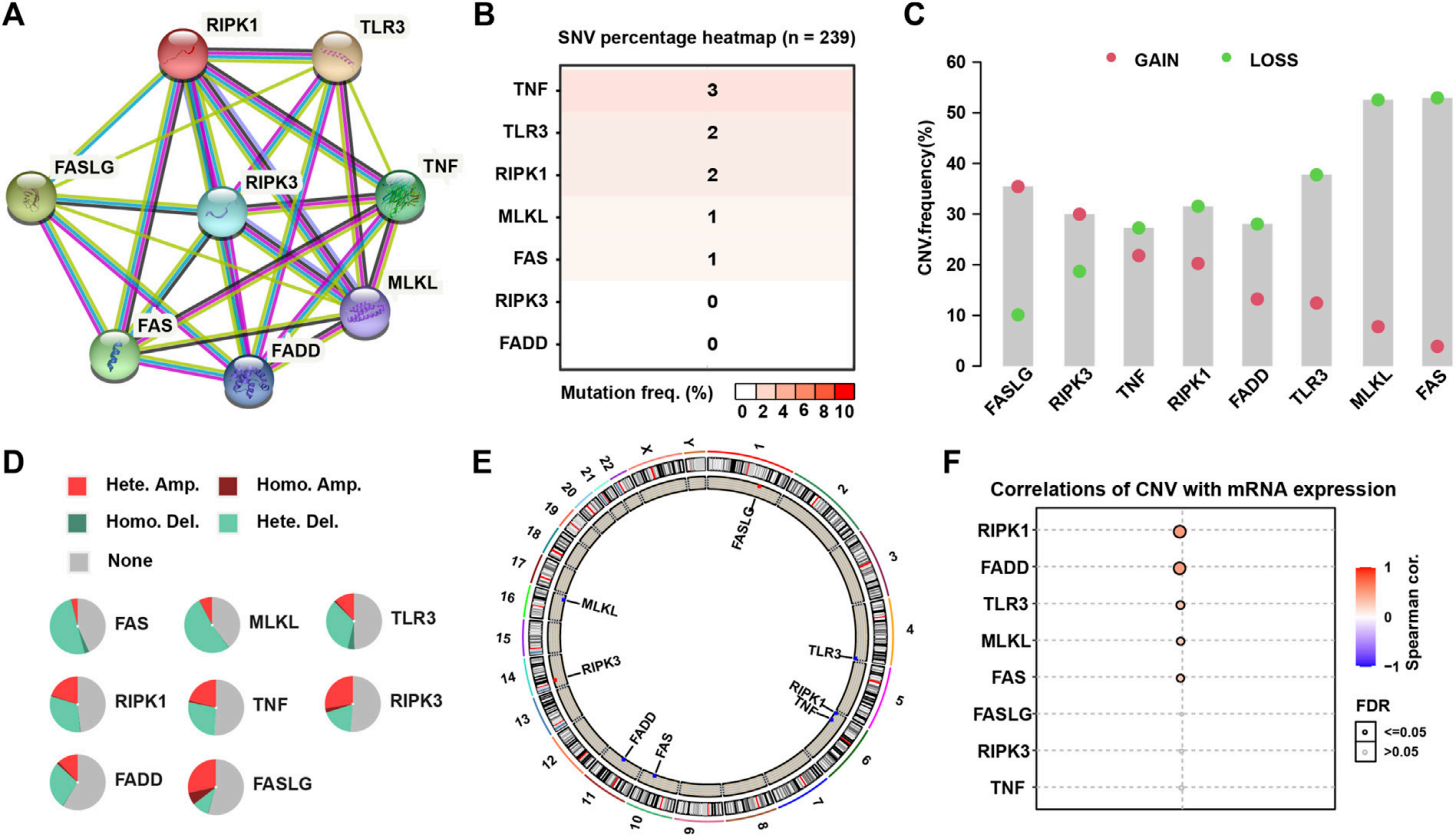
The PPI network and genetic alteration of necroptosis genes. **(A)** The PPI network of the eight necroptosis genes. **(B)** The SNV frequency of necroptosis genes in 239 SARC samples. **(C)** Frequencies of gain and loss CNV of necroptosis genes. **(D)** Pie plots presenting CNV percentages of necroptosis genes. **(E)** Chromosome locations of necroptosis genes. **(F)** The correlation between mRNA expression of necroptosis genes and their CNV.

The research flow chart was showed in Figure 1. The PPI network displayed intensive connections among the eight necroptosis genes where RIPK3 served as a central gene tightly connected with all the rest seven necroptosis genes (Figure 2A). We firstly explored the gene alternations including SNVs and CNVs of the necroptosis genes. The SNV frequency of necroptosis genes were generally low, in which TNF had the highest SNV frequency of 3% (Figure 2B). Of the CNV distribution, FASLG showed the highest frequency of amplification CNV (about 35%), while MLKL and FAS had the most proportion of deletion CNV of more than 50% (Figure 2C). In addition, hetezygous deletion and amplification CNV consisted of the majority (Figure 2D; Supplementary Figure S1). The chromosome localization of necroptosis genes were shown in Figure 2E. Spearman correlation analysis demonstrated that mRNA expression of the five genes (RIPK1, FADD, TLR3, MLKL, and FAS) was positively correlated with CNV (*p* < 0.05, Figure 2F). The above results suggested that a positive correlation between the expression of necroptosis genes and CNV profile.

### Identification of necroptosis subtypes based on the eight necroptosis genes

We merged the expression data of TCGA-SARC and GSE21257 datasets to analyze the correlation between the expression of necroptosis genes and the prognosis of SARC with correlation and univariate regression analysis. Apart from FADD, other seven necroptosis genes were all favorable factors, and RIPK3, TLR3, and TNF were significantly correlated with prognosis (*p* < 0.05, Supplementary Figure S2A). Kaplan-Meier survival analysis showed that SARC patients had longer overall survival with high expression of TNF, TLR3, PIPK3, PIPK1, MLKL, FASLG, and FAS (*p* < 0.05, Supplementary Figure S2B).

**FIGURE 3:**
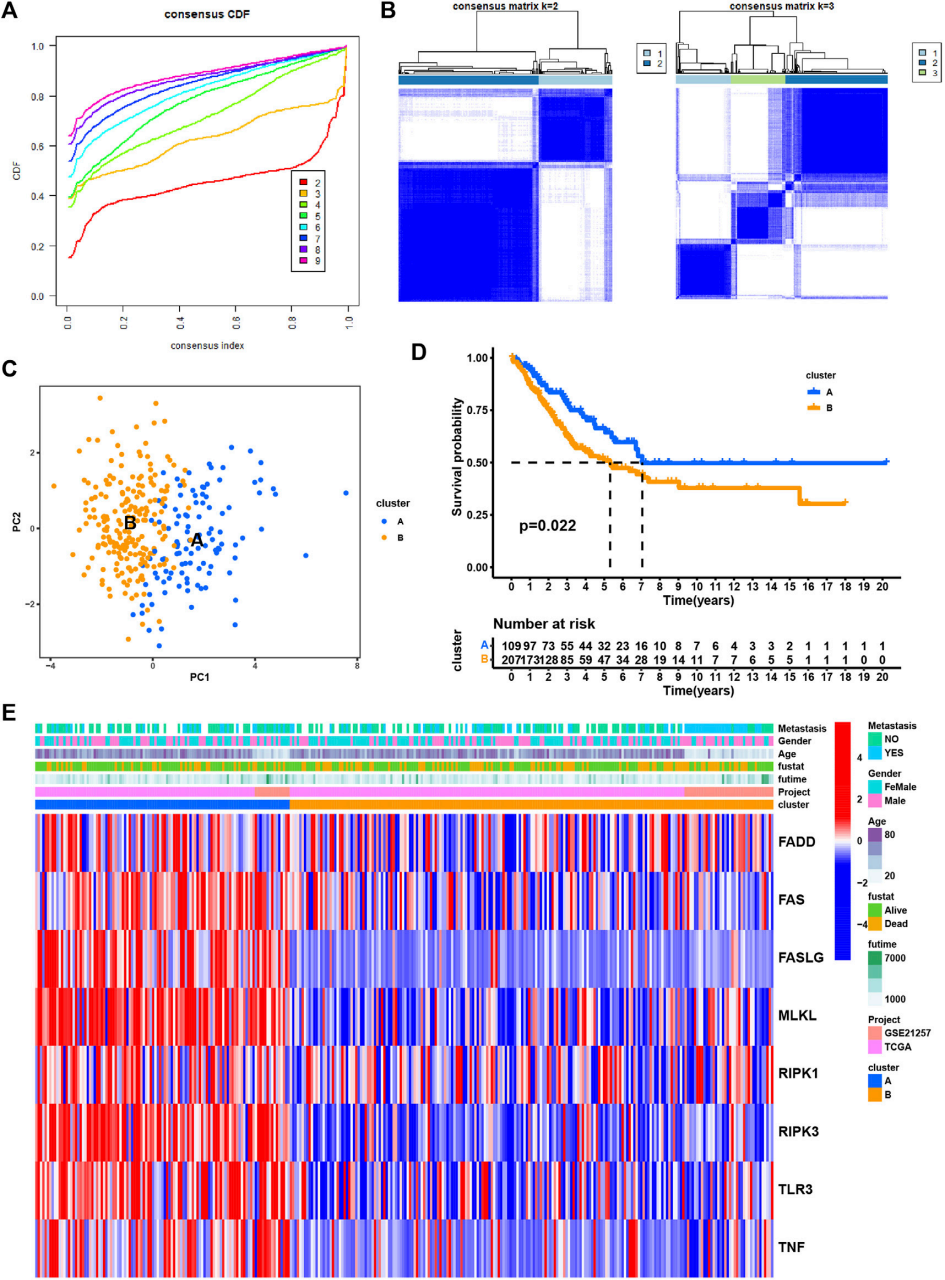
Identification of subtypes based on the eight necroptosis genes. **(A)** Cumulative distribution function (CDF) curve under different cluster number (K) **(B)** Consensus matrix heatmap defining different clusters (k = 2 and 3) and their correlation area. **(C)** PCA plot displaying the distribution of cluster A and **(B,D)** Kaplan-Meier survival analysis of SARC patients in two clusters. **(E)**. Distributions of clinical features and expression levels of necroptosis genes between two clusters.

**FIGURE 4:**
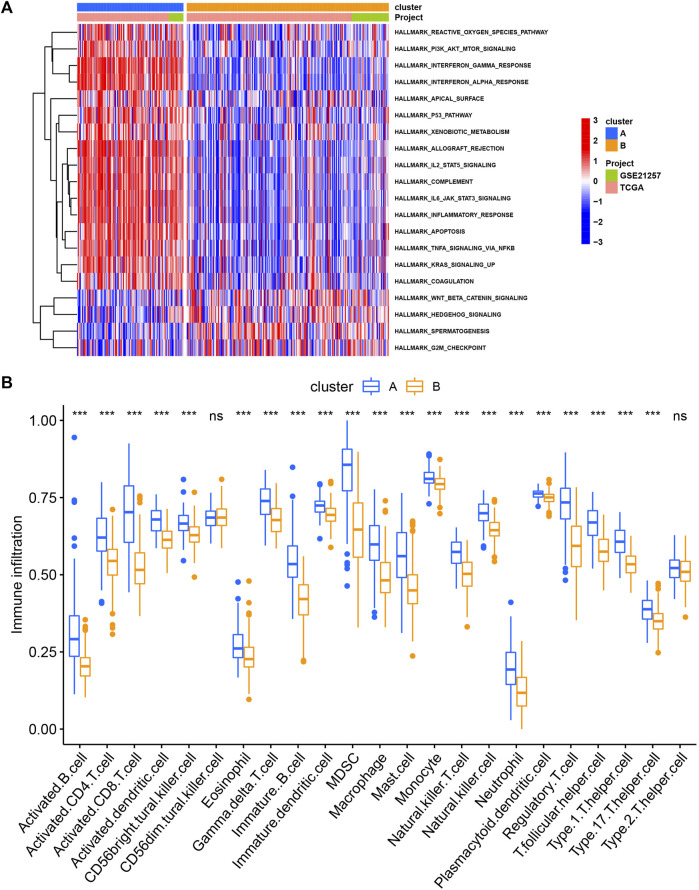
The different pathways **(A)** and immune infiltration **(B)** between cluster **(A)** and cluster **(B)** *p < 0.05, **p < 0.01, ***p < 0.001, ****p < 0.0001.

Next, we performed consensus clustering to cluster SARC patients based on the expression of necroptosis genes. SARC patients were clearly divided when cluster number was two in both consensus matrix and PCA visualization (Figures 3A–C). Two molecular subtypes (cluster A and cluster B) were determined and the prognosis of cluster A significantly outperformed that of cluster B (*p* = 0.022, Figure 3D). The distribution of expression of necroptosis genes grouped by clusters and clinical features revealed that cluster B had obviously lower expression than cluster A (Figure 3E), which was consistent with the result in Supplementary Figure S2B. Moreover, we analyzed the enriched pathways in the two subtypes using GSVA. In HALLMARK pathways, a number of immunity-related pathways such as interferon gamma response, interferon-alpha response, IL2-STAT5 signaling, IL6-JAK-STAT3 signaling, and inflammatory response, were significantly upregulated in cluster A (Figure 4A), those data imply that cluster A was more correlated with Tumorigenesis related pathways In Reactome pathways and KEGG pathways, we observed similar results (Supplementary Figure S3), suggesting that cluster A had a more active immune response than cluster B contributing to a favorable prognosis. We then estimated the infiltration level of 23 immune cells, and found that 21 of 23 immune cells, such as T cells, B cells, NK cells, Macrophage, Mast cell, and activated dendritic cells, were highly infiltrated in cluster A (*p* < 0.001, Figure 4B), which supported the above observation.

### Identification of necroptosis-related subtypes based on DEGs

**FIGURE 5:**
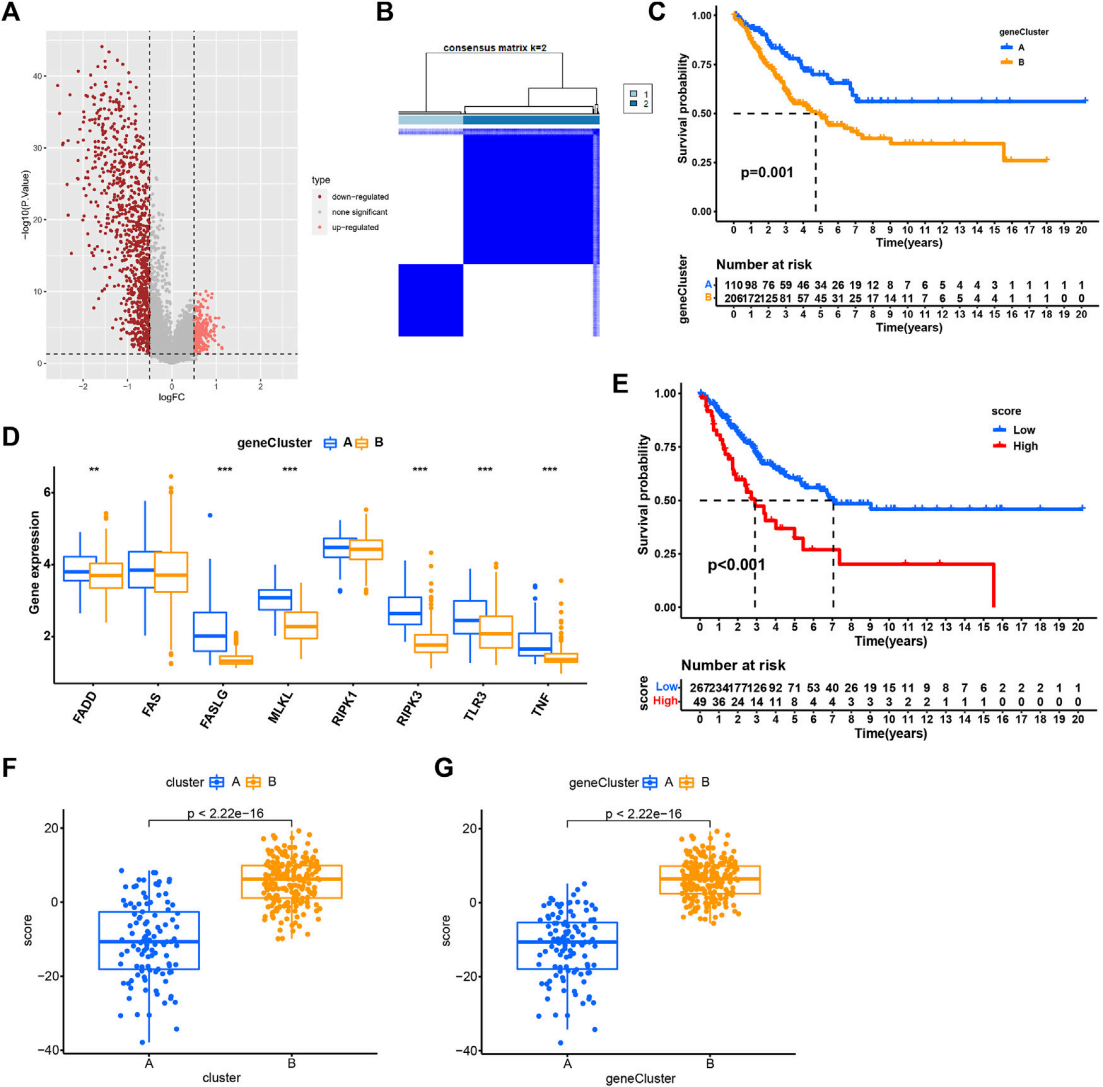
Identification of subtypes based on DEGs. **(A)** Volcano plot of 316 DEGs. **(B)** Consensus matrix heatmap when cluster number k = 2. **(C)** Kaplan-Meier analysis of SARC patients in two geneClusters. **(D)** The expression of necroptosis genes in two geneClusters. **(E)** Kaplan-Meier analysis of necroptosis score in SARC. **(F–G)** The necroptosis score in two clusters **(F)** and two geneClusters **(G)**. *p < 0.05, **p < 0.01, ***p < 0.001, ****p < 0.0001.

**FIGURE 6:**
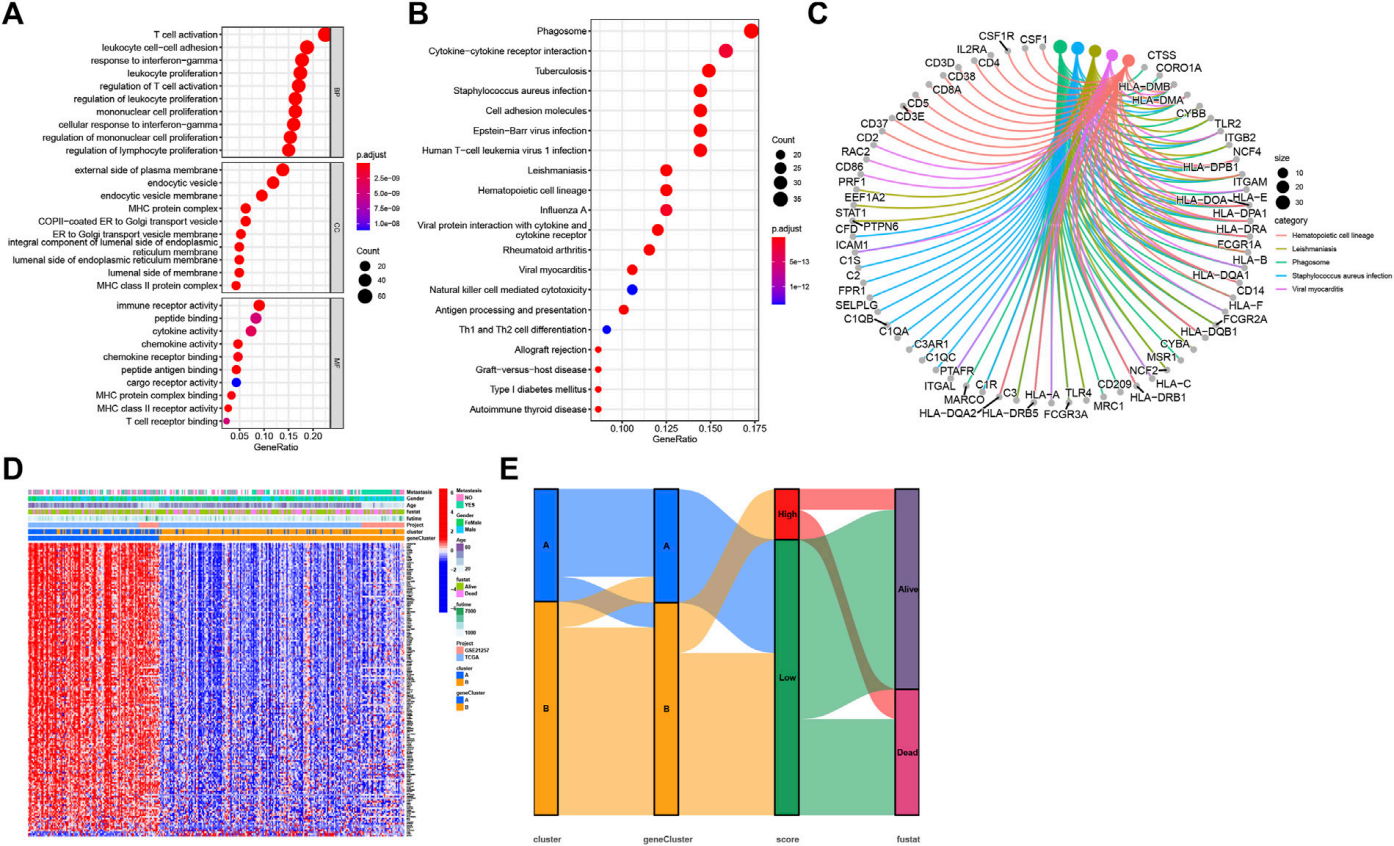
Functional enrichment analysis. **(A)** GO enrichment analysis of DEGs, including BP, CC, and MF. **(B)** The top 20 terms of KEGG results. **(C)** The top five terms of KEGG results with detailed DEGs. **(D)** The normalized expression of 173 DEGs grouping by geneClusters and the distribution of corresponding clinical features. **(E)** The sankey diagram visualized the correlation between cluster, geneCluster, necroptosis score, and survival status of SARC patients.

As cluster A and B exhibited significant differences on overall survival, tumor microenvironment, and enriched pathways, we next screened DEGs between the two subtypes (Figure 5A). 316 DEGs were filtered using “limma” package. Enrichment analysis of GO revealed that these DEGs were enriched in T cell activation, leukocyte proliferation, and cellular response to interferon-gamma in Biological Process (BP), external side of plasma membrane, MHC protein complex, and MHC class II protein complex in Cellular Component (CC), and immune receptor activity, cytokine activity, and chemokine activity in Molecular Function (MF) (Figure 6A). For KEGG pathways, immunity-related pathways were mostly enriched such as phagosome, cytokine-cytokine receptor interaction, and antigen processing and presentation (Figure 6B). The top five KEGG pathways with detailed DEGs were displayed (Figure 6C).

Univariate Cox regression analysis was performed to analyze ;the prognostic value of 316 DEGs, and 173 genes were found to be related to overall survival (*p* < 0.05) (Supplementary Table S2). To verify the importance of necroptosis on regulating these prognostic DEGs, unsupervised clustering was performed to divide patients into another two subtypes (geneCluster A and B) based on 173 prognostic genes (Figure 5B). Most of prognostic genes were overexpressed in geneCluster A when compared to geneCluster B (Figure 6D). SARC patients in geneCluster A had a more favorable survival than those in geneCluster B (*p* = 0.001, Figure 5C). In addition, we also observed the six necroptosis genes differentially expressing between two gene Clusters (*p* < 0.01, Figure 5D), suggesting that these prognostic genes may be involved in the regulation of necroptosis genes.

### Construction of necroptosis score

Given that two geneClusters had survival and expression differences, we then built a scoring system for predicting SARC prognosis. We established the necroptosis score based on the 173 prognostic genes using PCA algorithm. SARC patients were grouped into high- and low-necroptosis score groups according to PCA score. The patients with high necroptosis score had worse overall survival (*p* < 0.01, Figure 5E). In addition, cluster A and geneCluster A both had lower necroptosis score, which was in accordance with their prognosis (*p* < 0.0001, Figures 5F,G). The sankey diagram visualized the correlation between cluster, geneCluster, necroptosis score, and survival status (Figure 6E). Moreover, dead patients, metastatic patients, and female patients were more accumulated in the high-necroptosis score group, and they also showed a higher necroptosis score (Supplementary Figure S4).

**FIGURE 7:**
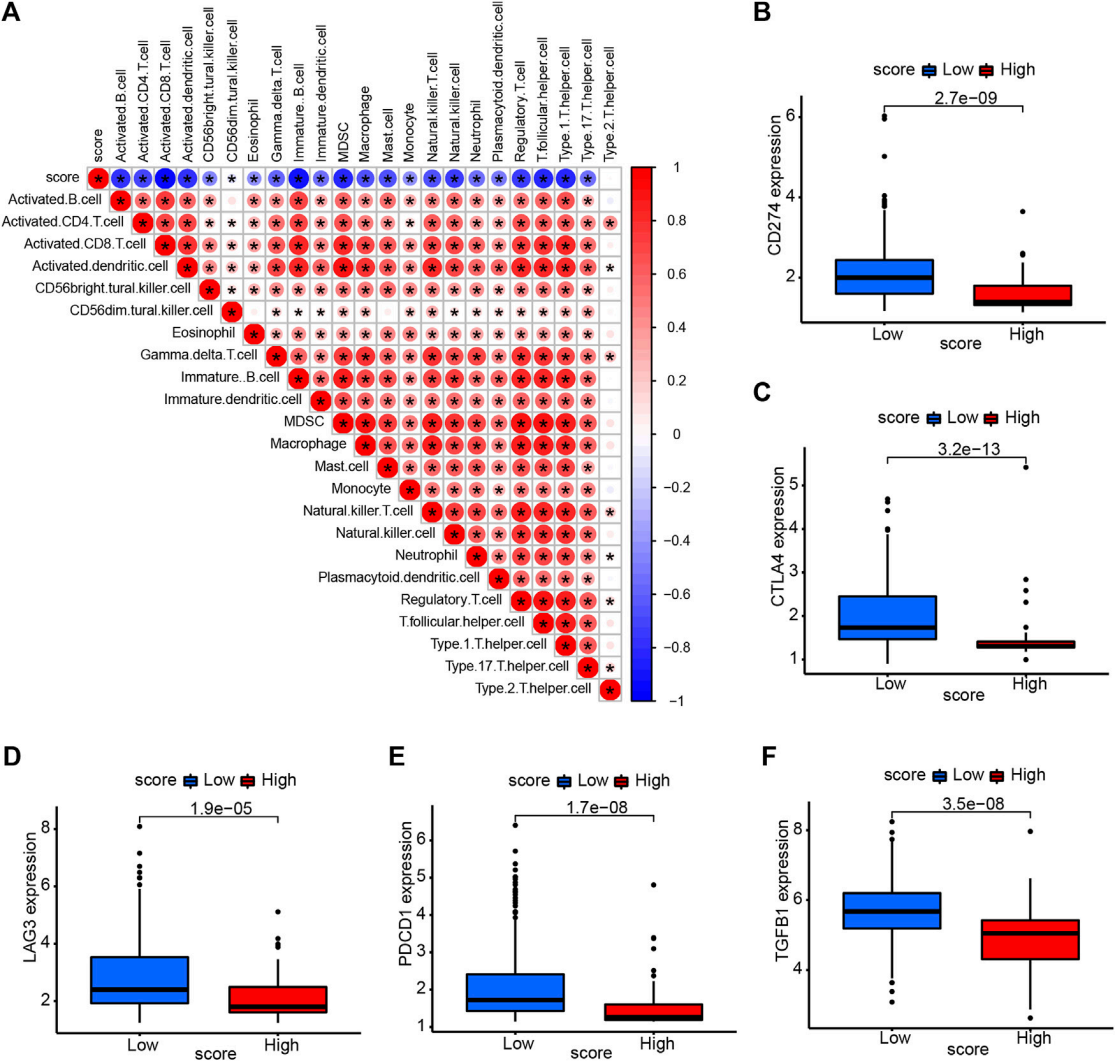
Association of necroptosis score and tumor microenvironment. **(A)** The correlation between necroptosis score and immune cell infiltration. Red color represents positive correlation, Blue color represents negative correlation. **(B–F)** The expression of immune checkpoints in high- and low-necroptosis score groups.

We also assessed the correlation between necroptosis score and tumor microenvironment. The necroptosis score was negatively correlated with the most of immune cells, indicating that higher necroptosis score had a lower immune cell infiltration level (Figure 7A). We selected some important immune checkpoints, including CD274, CTLA4, LAG3, PDCD1, and TGFB1, and evaluated their expression level in high- and low-necroptosis score groups. The result showed that low-necroptosis score group had higher expression levels of all five immune checkpoints (Figures 7B–F), suggesting that they may have different response to immune checkpoint blockade.

**FIGURE 8:**
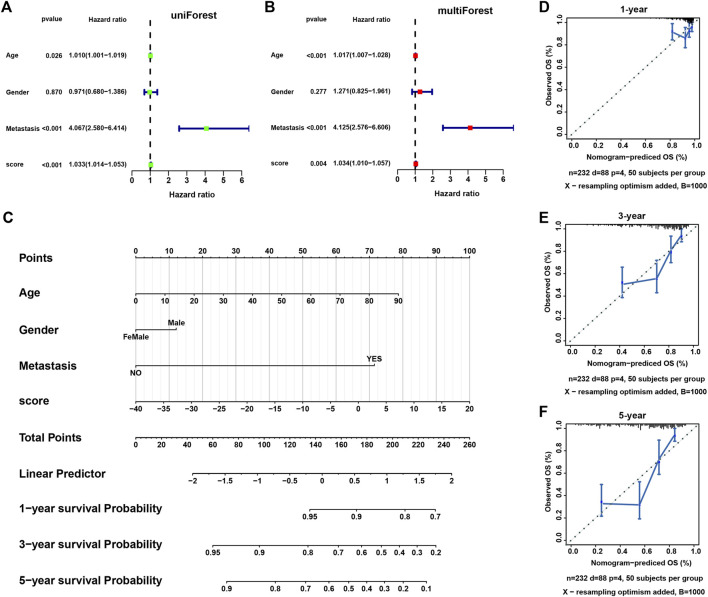
The construction of a nomogram model. **(A,B)** The univariate and multivariate Cox regression analysis of necroptosis score and other clinical features. **(C)** A nomogram based on necroptosis score and clinical features for predicting 1-, 3-, and 5-year survival. **(D–F)** The calibration plots showing the performance of the nomogram of predicting 1-, 3-, and 5-year overall survival.

Univariate and multivariate regression analysis showed that necroptosis score was an independent prognostic factor in SARC (Figures 8A,B). We further constructed a nomogram to predict the 1-, 3-, and 5-year overall survival of SARC patients (Figure 8C). The calibration of nomogram model was evaluated using calibration plots (Figures 8D–F).

### Drug resistance analysis of necroptosis genes

**FIGURE 9:**
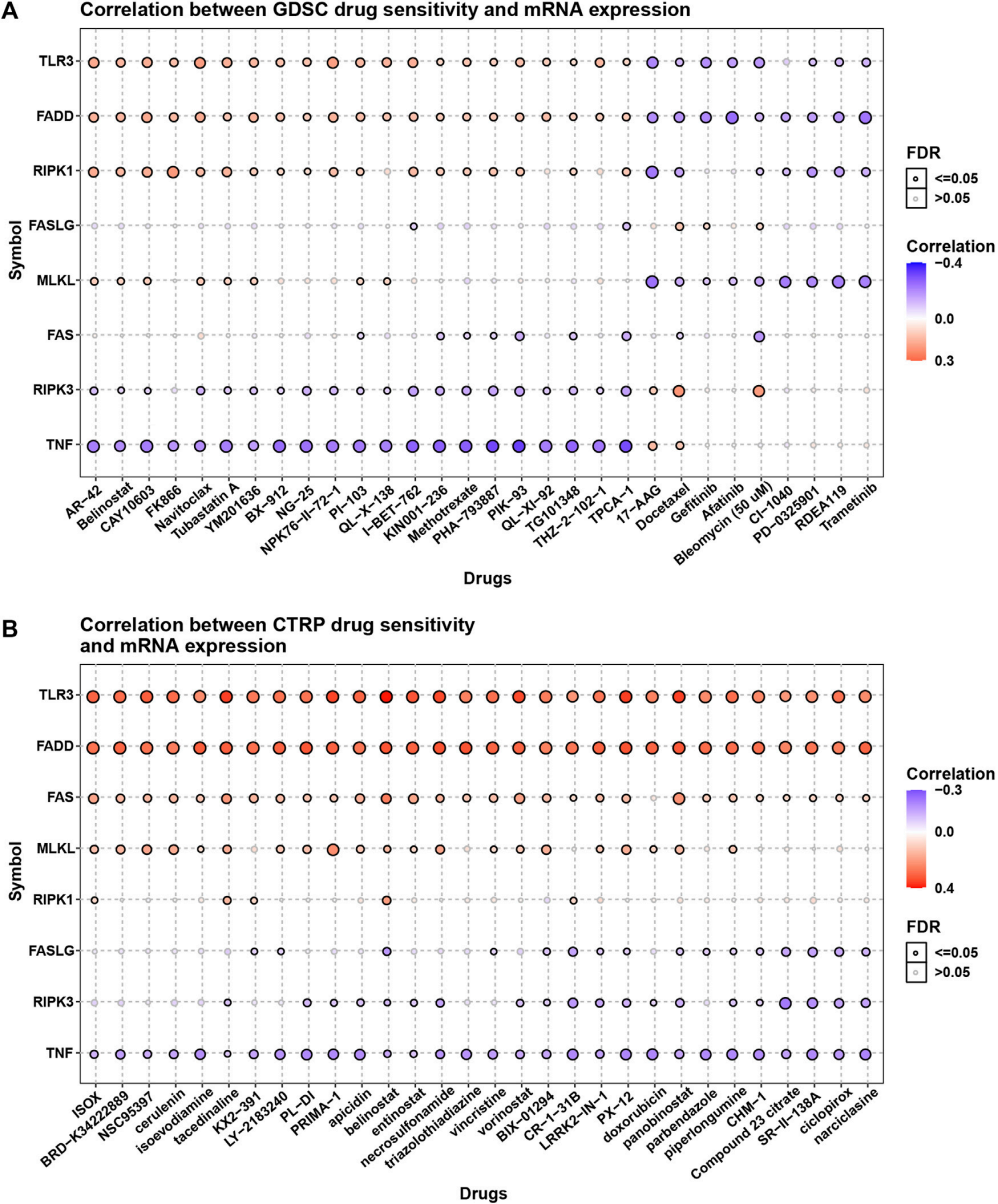
The association of necroptosis genes with efficiency of anti-tumor drugs. **(A)** The correlation between necroptosis score and IC50 of anti-tumor drugs based on GDSC database. **(B)** The correlation between necroptosis score and IC50 of anti-tumor drugs based on CTRP database.

The essential role of necroptosis genes in SARC have been shown, and we next explored the correlation of necroptosis genes with the efficacy of anti-tumor drugs. For the results based on GDSC data, we found that various genes have different sensitivities to different drugs (Figure 9A). For example, patients with high TNF expression may be sensitive to AR-42, Belinostat, and CAY10603. For the results based on CTRP data, we observed that patients with high expression of TNF may be sensitive to most anti-tumor drugs, such as ISOX, BRD-K34222889, and NSC95397 (Figure 9B).

## Discussion

The occurrence of cancer is closely related to cell death, and in addition to apoptosis, necroptosis is similarly associated with the development of cancer. However, studies have proven that necroptosis plays a dual role in cancer progression and progression (Wang et al., 2018). Among them, targeting necroptotic proteins has dual effects on tumor initiation and progression. Generally, studies agree that the dysfunction of necroptosis is associated with tumor initiation and progression. For example, RIPK3 expression is significantly down-regulated in patients with acute myeloid leukemia (AML). Reduction of RIPK3 decreases hematopoietic cell death, which is associated with the development of AML (Nugues et al., 2014). In addition, low expression of MLKL is related to decreased overall survival in colon cancer patients after surgery (Li et al., 2017). MLKL is also downregulated in pancreatic cancer and cervical squamous cell carcinoma, where low levels of MLKL in plasma predict poor prognosis in pancreatic and ovarian cancer (Ruan et al., 2015; Seldon et al., 2016). However, the role of necroptosis has not been comprehensively studied in SARC.

In our study, a total of eight necroptosis genes were enrolled. Based on the expression of necroptosis genes, we identified two subtypes (cluster A and cluster B) of SARC. The expression of necroptosis genes was significantly higher in cluster A than in cluster B. Compared to the patients with cluster B, the patients with cluster A had longer overall survival and reduced metastasis, suggesting that high expression of necroptosis genes may contribute to favorable prognosis. In addition, abundant genomic alternations including gain and loss of CNV were observed in the necroptosis genes. Especially, the mRNA expression of RIPK1 and FADD were positively associated with their CNVs. It could be speculated that CNV was one of factors resulting in the dysregulation of necroptosis in sarcoma.

To understand the potential mechanism of necroptosis in SARC, the enriched pathways of Reactome, HALLMARK and KEGG were all assessed in the two clusters. Immune-related pathways were obviously activated in cluster A, such as interferon gamma response, interferon alpha response, T cell receptor signaling pathway, and chemokine signaling pathway. Not surprisingly, higher immune cell infiltration was found in cluster A, indicating a close correlation between necroptosis and tumor microenvironment. Necroptosis is involved in immunogenic cell death in TME (Scarpitta et al., 2021). Manipulation of necroptosis opens a new way to induce tumor cell death and to trigger an efficient stimulation of adaptive anti-tumor T cells (Snyder et al., 2019). Furthermore, enrichment analysis on the DEGs between cluster A and B also illustrated that immune-related pathways, such as cytokine-cytokine receptor interaction, cell adhesion molecules, and antigen processing and presentation pathways, were significantly enriched, which supported the conclusion that necroptosis-induced tumor cell death was a promising strategy for cancer treatment.

Thus, we constructed a necroptosis score to evaluate the risk of patients. We performed univariate cox regression analysis to identify the prognostic value of 316 DEGs and filtered 173 genes related to overall survival (*p* < 0.05). The necroptosis score was established based on the 173 prognostic genes using PCA algorithm. Patients with high necroptosis score have worse survival status. The correlation of necroptosis score with immune cell infiltration indicated that necroptosis score was negatively associated with immune cell infiltration level.

Immune checkpoint is one of the predictors of the efficacy of immune checkpoint inhibitor (ICI) treatment (Pinato et al., 2019) (Liu et al., 2019). We found that immune checkpoints, such as CD274, CTLA4, LAG3, PDCD1, and TGFB1, were high-expressed in low-necroptosis score group. These results indicated that patients with high necroptosis score may be more resistant to ICI treatment.

In conclusion, we conducted a comprehensive analysis of necroptosis genes and revealed its extensive regulatory mechanisms affecting tumor immune microenvironment, clinical features and the prognosis of SARC patients. We also constructed a necroptosis score and determined its reliability in predicting SARC prognosis and ICI treatment efficacy. These findings highlighted the important clinical significance of necroptosis and provided a new idea for guiding the personalized treatment strategy of SARC patients.

## Data Availability

The datasets presented in this study can be found in online repositories. The names of the repository/repositories and accession number(s) can be found in the article/Supplementary Material.
