# Supplementary figures and images for: Characterizing the prognostic and therapeutic value of necroptosis in sarcoma based on necroptosis subtypes
Source: Front Genet. 2022 Sep 27;13:980209. doi: 10.3389/fgene.2022.980209 (PMC9552825; doi:10.3389/fgene.2022.980209)

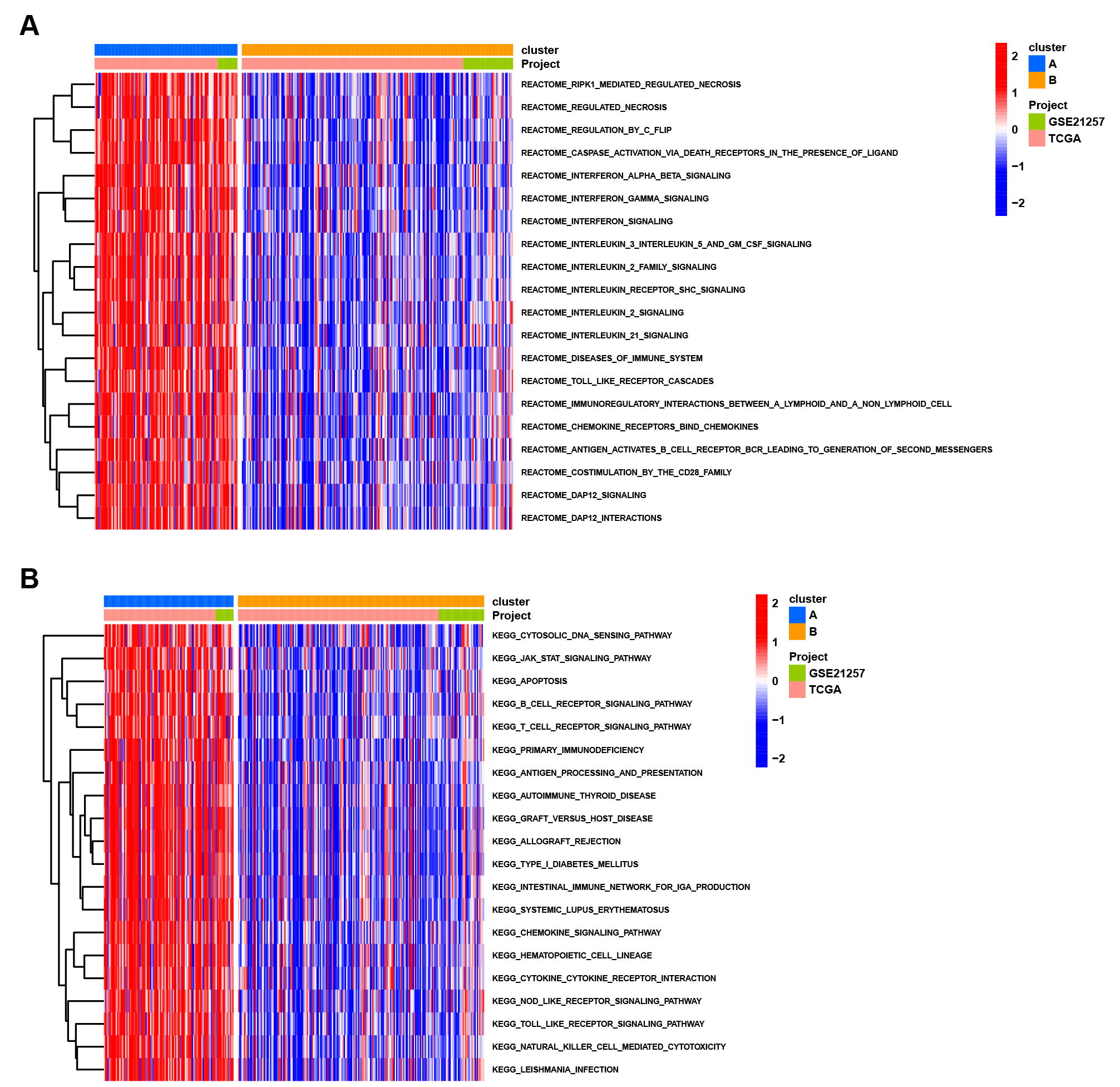

Supplement: Supplementary file 2 [file Image3.JPEG]

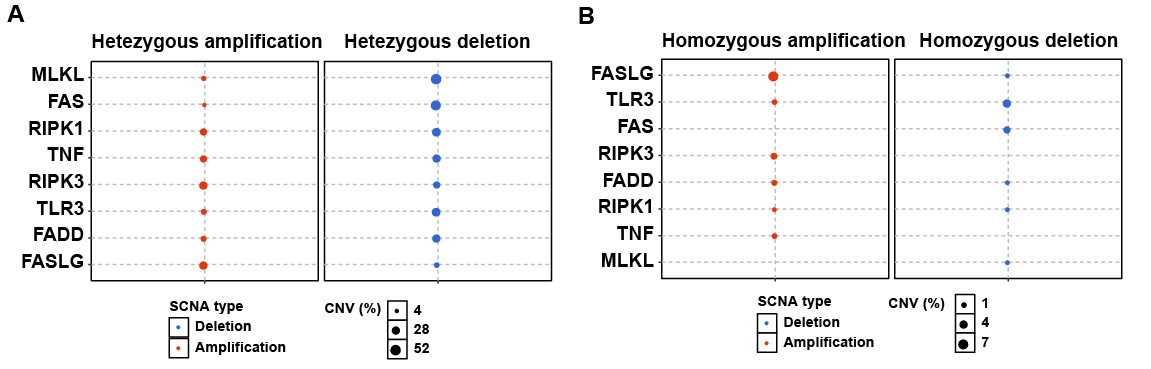

Supplement: Supplementary file 4 [file Image1.JPEG]

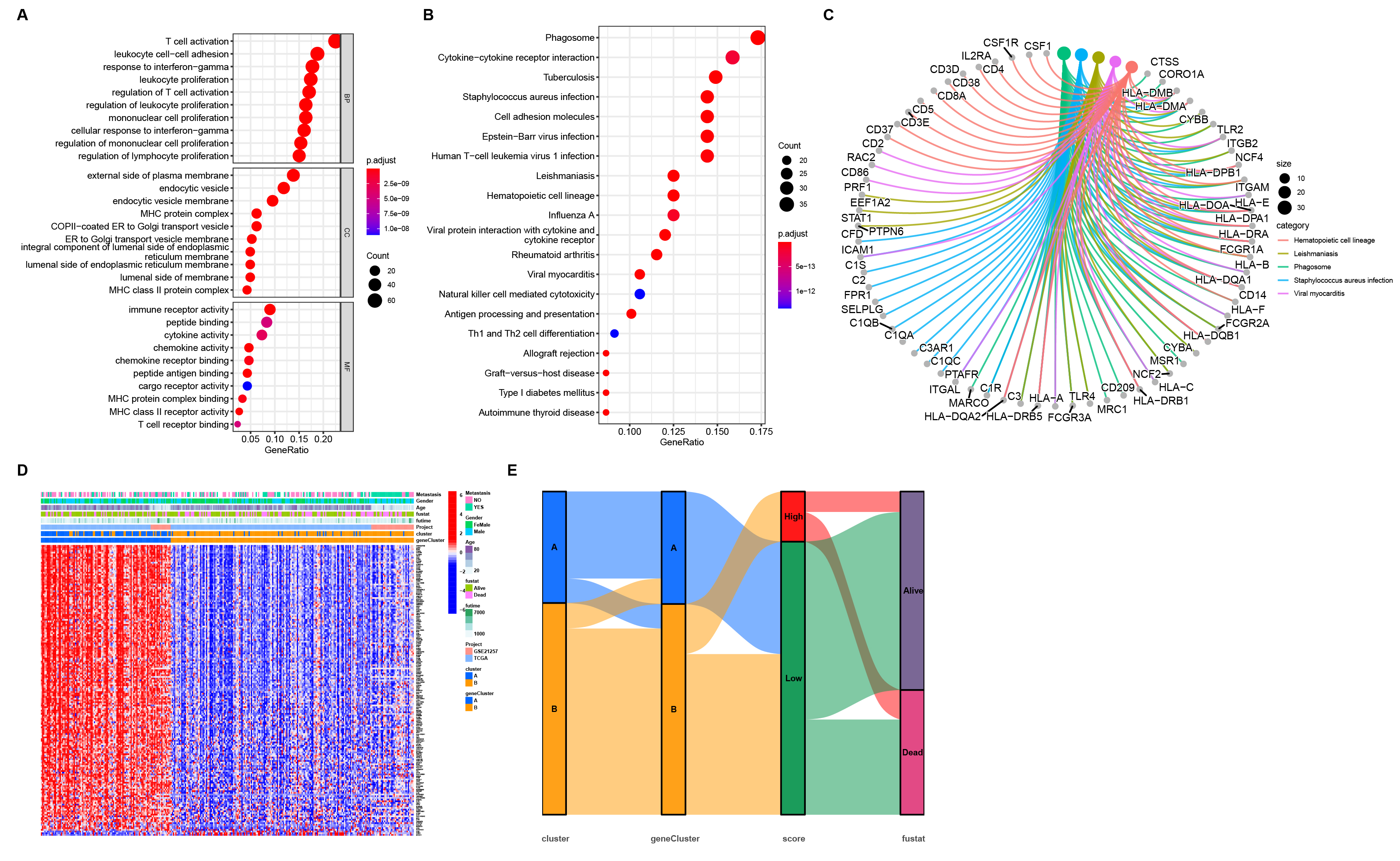

Supplement: Supplementary file 5 [file Image4.JPEG]

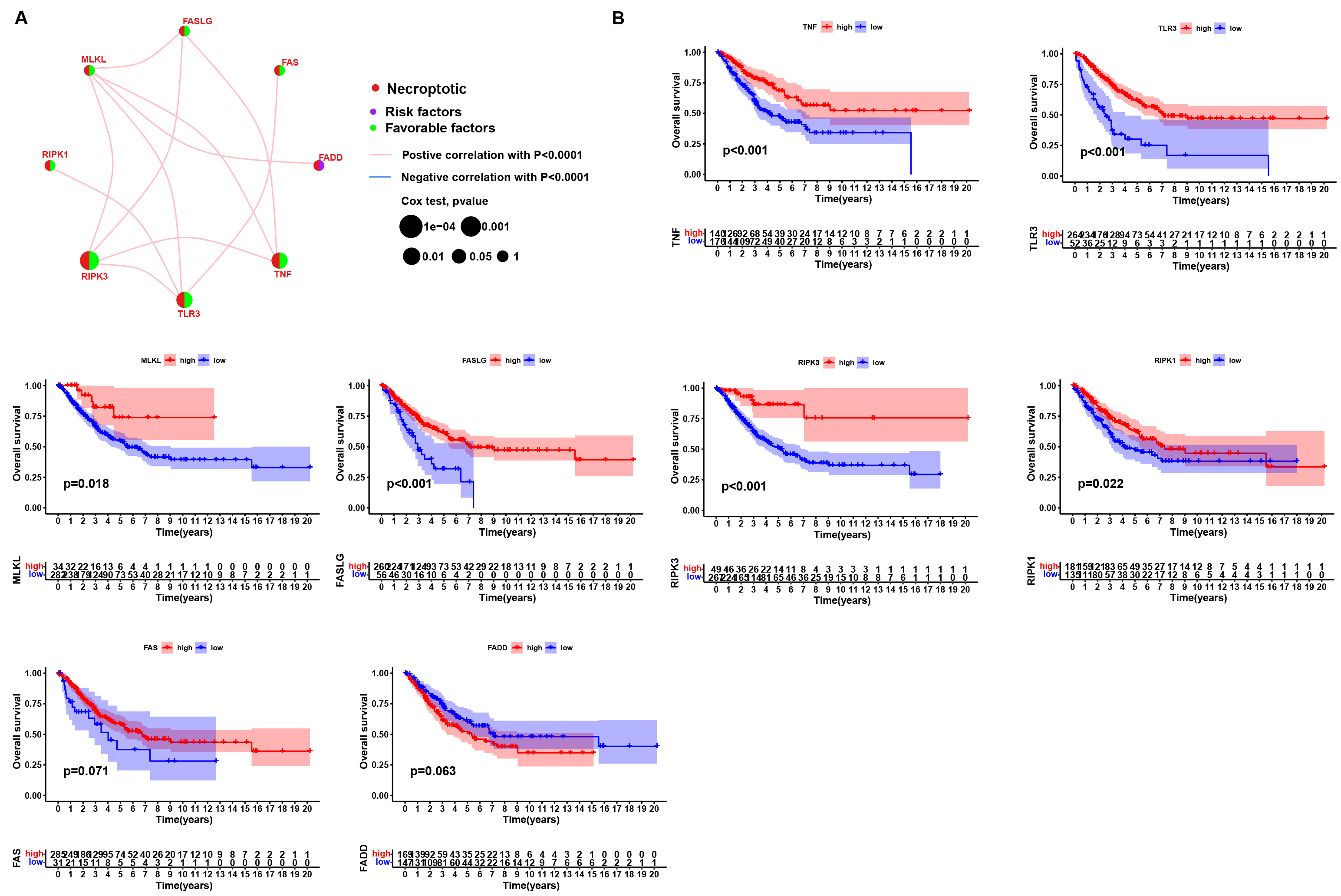

Supplement: Supplementary file 6 [file Image2.JPEG]

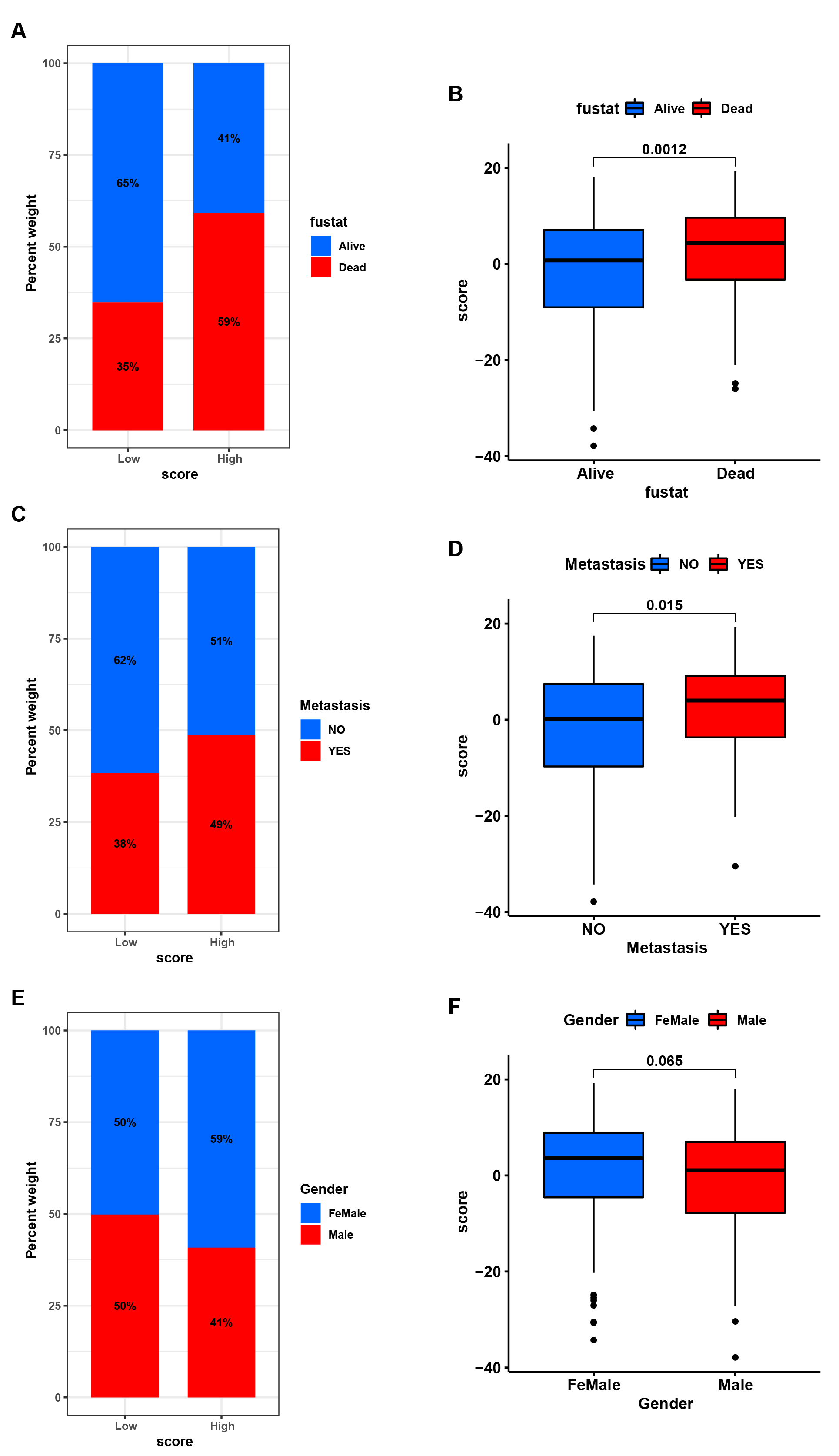

Supplement: Supplementary file 7 [file Image5.JPEG]
