# Supplementary material for: Characterizing the prognostic and therapeutic value of necroptosis in sarcoma based on necroptosis subtypes
Source: Front Genet. 2022 Sep 27;13:980209. doi: 10.3389/fgene.2022.980209 (PMC9552825; doi:10.3389/fgene.2022.980209)
Supplement: Supplementary file 8 [file Table1.DOC]

Data analyzed in this study, please see:

https://www.jianguoyun.com/p/Ddvpx_AQ9anaChjS0MgEIAA
